# Giant pandas’ staple food bamboo phyllosphere fungal community and its influencing factors

**DOI:** 10.3389/fmicb.2022.1009588

**Published:** 2022-09-30

**Authors:** Liwen Kang, Wei Luo, Qinglong Dai, Hong Zhou, Wei Wei, Junfeng Tang, Han Han, Yuan Yuan, Juejie Long, Zejun Zhang, Mingsheng Hong

**Affiliations:** ^1^Liziping Giant Panda’s Ecology and Conservation Observation and Research Station of Sichuan Province (Science and Technology Department of Sichuan Province), China West Normal University, Nanchong, China; ^2^Key Laboratory of Southwest China Wildlife Resources Conservation (Ministry of Education), China West Normal University, Nanchong, China; ^3^Liziping National Nature Reserve Administration, Ya’an, China

**Keywords:** giant panda, staple food bamboo, phyllosphere fungi, metagenome, ARGs, Liziping NR

## Abstract

Giant pandas have developed a series of foraging strategies to adapt to their special bamboo diets. Although bamboo is an important food resource for giant pandas in Liziping National Nature Reserve (Liziping NR), China, there are relatively few studies on their phyllosphere fungal community and its influencing factors. Herein, we used ITS1 amplification and metagenomic sequencing to analyze the phyllosphere fungi diversity and functions (KEGG, CAZyme, and antibiotic resistance gene) and explore the influencing factors for the three giant pandas foraging bamboo species (*Arundinaria spanostachya*, AS; *Yushania lineolate*, YL; and *Fargesia ferax*, FF) over different seasons (spring vs. autumn) in Liziping NR, China. We found that Ascomycota and Basidiomycota were the most dominant phyla in the bamboo phyllosphere. The alpha diversity (e.g., the Sobs index and Shannon index) was relatively higher in autumn samples than in spring samples, and the community structure differed significantly between the three bamboo species in spring and autumn. Some biotic and abiotic variables (e.g., the elevation and mean base diameter of bamboo) significantly influenced the abundance, diversity, and community structure of the bamboo phyllosphere fungal community. Moreover, the functional analysis showed the differences in the glycoside hydrolase community and antibiotic resistance gene (ARG) profile between spring and autumn samples. Co–occurrence network modeling suggested that AS phyllosphere fungal communities in autumn employed a much more complex network than that in spring, and the abundance of multidrug, tetracycline, and glycopeptide resistance genes was high and closely correlated with other ARGs. These results indicate that fungal community’s abundance, diversity, and community structure are mainly affected by the season, host species, and elevation. The season and host species are major factors affecting the biological functions (KEGG and CAZyme), ARGs, and interactions between sympatric bacterial and fungal communities in bamboo phyllosphere. This integrated study can provide a reference basis for the seasonal management of bamboo resources foraged by wild giant pandas, and predict the risk of antibiotic resistance in bamboo phyllosphere fungal flora in Liziping NR (Xiaoxiangling mountains), China.

## Introduction

The giant panda (*Ailuropoda melanoleuca*) belongs to the order carnivore and has a typical carnivore gastrointestinal tract. Fossil evidence has shown that the giant panda at least 7 Myr ago started to forage bamboo (Jin et al., 2007; Wang X. M. et al., 2022). As the umami taste receptor gene *Tas1r1* had lost its function and other reasons by about 4.2 Myr ago (Zhao et al., 2010), it is clear that giant pandas had already completed their dietary switch by about 2.0–2.4 Myr ago (Jin et al., 2007).

To adapt to the special bamboo diet, giant pandas have developed a series of foraging strategies, including the evolution of pseudo-thumbs, seasonal foraging, feeding point selection, habitat selection, etc. (Endo et al., 1999; Wei et al., 1999; Hong et al., 2015, 2016; Hu et al., 2017). However, in different seasons and mountain regions, giant pandas feed on different parts of bamboo and different kinds of bamboo; however, bamboo leaves are still their predominant food throughout the year (Wei et al., 2015). For example, giant pandas forage bamboo leaves most of the year except May–July in the Qinling Mountains. (Wu et al., 2017). Despite the fact that giant pandas only feed on bamboo, they have not evolved any genome-encoding enzymes specific for cellulose digestion (Hu et al., 2017). Thus, bamboo leaves are an important reason driving the composition and changes of intestinal flora of giant pandas; imbalances in this intestinal flora can lead to gastrointestinal disease, which is the most common cause of death in captive and wild giant pandas (Tun et al., 2014).

As an important food source for giant pandas, bamboo leaves have different nutrient and microbial compositions as compared with bamboo shoots, bamboo stems, and branches (Wei et al., 2015; Wu et al., 2017; Long et al., 2021). The health status of bamboo leaves, including the differences in seasonal nutrition and microorganisms in bamboo phyllosphere, could be the reason for the variations in giant pandas’ seasonal activities and physiology. Using the traditional pathology method, Xu et al. (2014) found that the microbial community composition of phyllosphere differed among different bamboo species, and microbial groups showed seasonal differences. There were also differences in species composition and frequency of endophytic bacteria and fungi in bamboo leaves (Helander et al., 2013), while endophytic fungi in *Phyllostachys heteroclada* differ in the leaf and branch (Zhou et al., 2017). Using high-throughput amplicon sequencing, Jin et al. (2020) found that the richness and diversity of bacteria and fungi differed significantly between bamboo species.

The phyllosphere is one of the most diverse ecosystems on earth (Lindow and Leveau, 2002; Corinne et al., 2016), inhabited by bacteria, archaea, fungi, and protists (Arnold et al., 2003; Lindow and Brandl, 2003). Bacteria are the most abundant colonizers in leaves. However, phyllosphere fungal colonizers are incredibly diverse (Lindow and Brandl, 2003; Finkel et al., 2011; U’Ren et al., 2012). During the growing season, bacteria dominate, yeasts followed, and filamentous fungi accounted for the least (John and Robin, 2000; Redford and Fierer, 2009). The phyllosphere community composition is significantly impacted by biological and abiotic factors, such as the host species, season, temperature, humidity, host attributes, etc. (John and Robin, 2000; Lindow and Brandl, 2003; Paulino-Lima et al., 2013; Kembel et al., 2014; Copeland et al., 2015).

As an important part of the ecosystem, phyllosphere microorganisms affect plant surface properties (Lindow and Brandl, 2003), which have a key role in the degradation of organic matter, nutrient conversion, and energy flow (Rastogi et al., 2013; Zhao et al., 2013; Horton et al., 2014). Phyllosphere fungi, which can prevent herbivores from eating plants, protect plants from all kinds of pathogens and also make the plant tolerant against abiotic stress (Rehman et al., 2021). The ability to degrade cellulose is widespread in fungi, and is particularly well represented in Ascomycota and Basidiomycota (Boer et al., 2005). In a paddy environment, Fan et al. (2019) found an indigenous fungus, *Aspergillus cvjetkovicii*, which exerted an effect of strong antagonism on *Magnaporthe oryzae* and a promoting effect on rice. Zahn and Amend (2019) found that phyllosphere fungi alter the timing and allocation of reproduction in *Arabidopsis*, resulting in later flowering and higher seed quality. However, with the abuse of antibiotics and the rapid spread and development of antibiotic resistance, antibiotic resistance has become one of the world’s greatest public health dangers (Chen et al., 2019; Pei et al., 2019). Unfortunately, phyllosphere is increasingly known as reservoirs of antibiotic resistance genes (Sun et al., 2021).

Most microorganisms do not exist in isolation, but live in the same space and share common substrates; these common substrates lead to either synergistic or antagonistic interactions, which directly or indirectly affect the growth of host plants and the absorption and utilization of nutrients (Mille-Lindblom et al., 2006; Schmidt et al., 2015; Ma et al., 2020). The interaction of bacteria and fungi can change soil structure and fertility, improve plant adaptability, and enhance the absorption and utilization of plants for nutrients and water (Porras-Soriano et al., 2009; Tang et al., 2020). Bacteria and fungi also work together to promote the rumen digestion of grass (Lee et al., 2000).

As mentioned above, only three studies have been conducted on the phyllosphere microflora of giant pandas’ staple food, bamboo (Helander et al., 2013; Jin et al., 2020; Long et al., 2021). Even fewer have been conducted on the phyllosphere microorganisms of bamboo leaves. Our previous research has focused on environmental factors influencing phyllosphere bacterial communities in bamboo in Liziping National Nature Reserve (Liziping NR), Sichuan, China (Long et al., 2021). According to the results of the Fourth National Giant Panda Survey, there are 22 wild giant pandas in the reserve (Sichuan Forestry Bureau, 2015). As of 2019, 1 rescued giant panda and 9 captive pandas were released into the reserve, alongside 8 surviving giant pandas (Hong et al., 2019). In this study, therefore, we focus on the phyllosphere fungal communities in giant pandas’ staple food bamboo and their influencing factors using next-generation sequencing (NGS) technology (ITS1 region amplicon sequencing and whole-genome shotgun sequencing). This study aimed to (i) compare interspecies differences and seasonal variations in the composition, diversity, and community structure of phyllosphere fungal communities in three bamboo species (*Arundinaria spanostachya*, AS; *Yushania lineolate*, YL; and *Fargesia ferax*, FF); (ii) explore the ecological factors that influence the phyllosphere fungal community changes; (iii) investigate the interactions between fungi and bacteria in bamboo phyllospheres using co-occurrence network analysis; (iv) reveal the differences in biological functions (KEGG and carbohydrate-active enzyme functions, CAZyme) of AS phyllosphere fungi between spring and autumn; and (v) obtain antibiotic resistance gene (ARG) abundances of the AS phyllosphere fungal community and their differences between spring and autumn, determining their interactive relationship. Our research results could provide a reference basis for the restoration, protection, and management of bamboo resources in wild giant panda habitats.

## Materials and methods

### Study area

**Figure 1:**
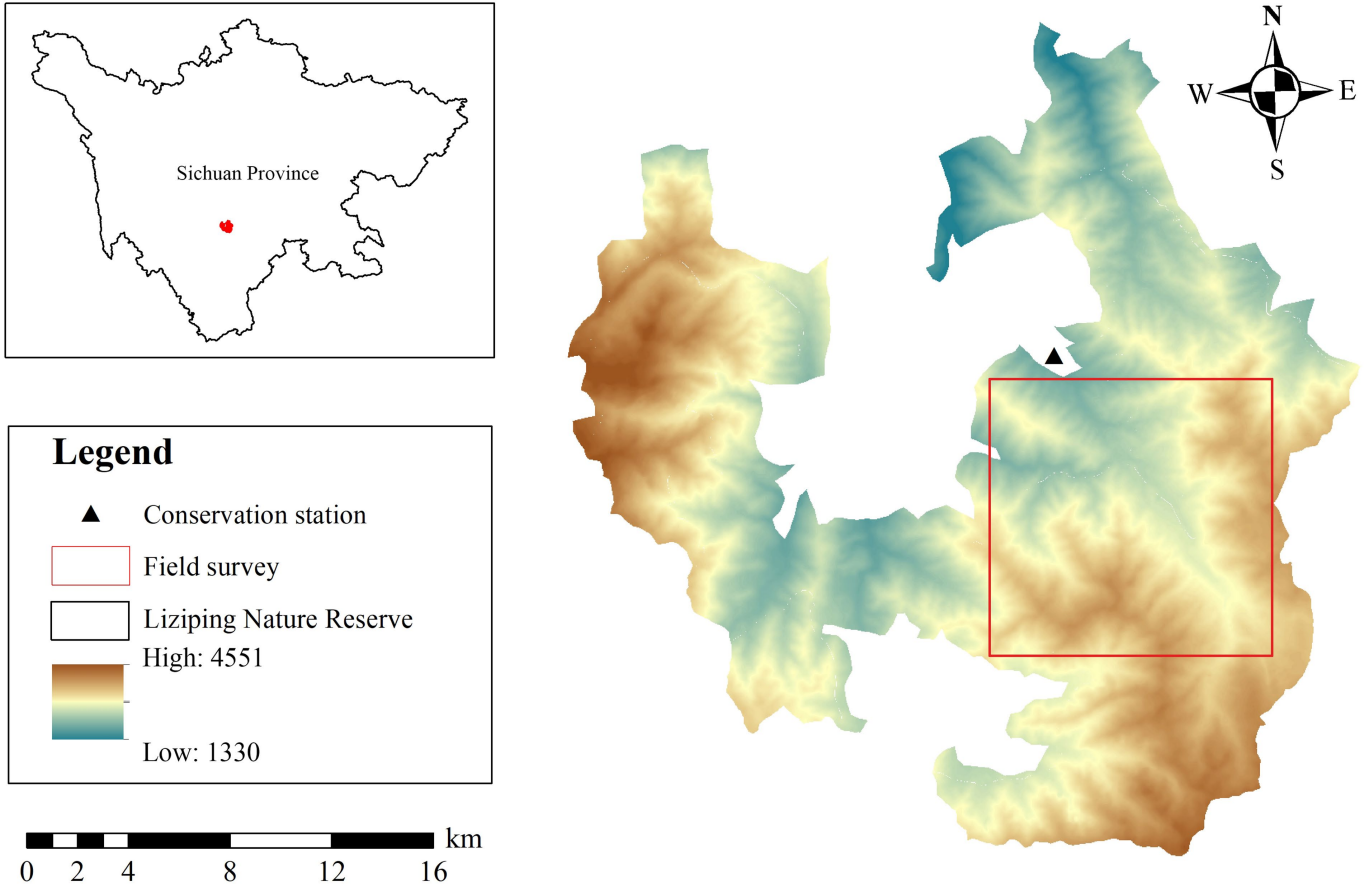
Study area in Liziping National Nature Reserve, China.

Liziping NR (28°51′02″–29°08′42″N, 102°10′33″–102°29′07″E) is situated in Shimian County, Sichuan Province, China (Figure 1), and lies in the southwest region of the Sichuan Basin; the largest tributary of the Minjiang River; and the southeast region of the Gongga Mountains. The reserve covers an area of about 47,885 hm^2^; the elevation of the reserve is from 1,330 m to 4,551 m. The annual mean temperature and annual mean precipitation are 11.7°C–14.4°C and 800–1,250 mm, respectively. As the altitude increases, the vegetation types of the reserve transition from evergreen broadleaved forest and evergreen broadleaved mixed forest to coniferous and deciduous broadleaved mixed forest and evergreen coniferous forest, and subsequently, to alpine shrub and alpine meadow (Hong et al., 2015). AS, YL, and FF are the three main species foraged by giant pandas in the reserve. The most widely distributed species is AS, accounting for 38.08% of the total area of bamboo for giant pandas in this mountainous region (Sichuan Forestry Bureau, 2015). YL and FF account for 28.02% and 12.48% of giant pandas’ diets, respectively (Sichuan Forestry Bureau, 2015). AS is mainly distributed in regions higher than 2,500 m above sea level, and YL and FF are mainly distributed below 2,800 m above sea level. AS was the main food year-round, YL was partial food for winter, and FF was occasional food (Xie and Hong, 2020; Long et al., 2021).

### Experimental design

Field surveys and sampling were conducted in May (spring) and October (autumn) in 2020 (Figure 1). First of all, 4 transect lines were set up in AS, YL, and FF forests, respectively. The transect lines were set along the elevation gradient, and each transect line was separated by more than 200 m. Second, 3–5 survey plots (20 m × 20 m) were set on each transect line, and adjacent survey plots with an altitude distance of more than 50 m were arranged on the same transect. The bamboo species, latitude and longitude, altitude, tree layer, shrub layer, and other related variables were recorded and measured in each survey plot. Then, 1 bamboo plot (1 m × 1 m) was set at the central point of each survey plot and 5 m east and south of the central point, and the related variable data of bamboo layers were measured and recorded (Supplementary Table S1).

### Sample collection and DNA extraction

For each survey plot, no <200 g of mixed bamboo leaves was collected using sterile gloves. Samples were immediately transported to the laboratory within 2 h after collection and stored in a −20°C freezer. Every 200 g sample was sterile transferred to a plastic bag (24 cm × 35 cm) containing 200 ml of sterile precooled TE-buffer (10 mM Tris, 1 mM EDTA, and pH 7.5); subsequently, 0.05% Tween-80 was added (Hong et al., 2017). The samples were cleaned to collect microbiota; each sample was shaken, eddy current, and ultrasonically stirred in a TE-buffer for 5 min, and the plastic bags were then stored in ice water (at ~4°C) for each treatment step. An ultrasonic scrubber with a frequency of 40 kHz (Shanghai Kudos Instrument Co., Shanghai, China) was used to remove microorganisms from bamboo leaves. The leaves were filtered through three layers of sterile nylon mesh for cell suspension. After filtration, the cell suspension was placed in four 50 ml tubes and centrifuged at 4°C for 15 min with the rate of 2,200 × *g* to remove the supernatant and obtain the fungus slime. Multi-tube mycelium was collected in a 2.0 ml reaction tube and washed twice with TE-buffer. The mud was immediately frozen at −80°C until DNA was extracted.

The DNA was extracted using the E.Z.N.A.TM Soil DNA Kit (Omega, Norcross, GA, United States) using the manufacturer’s specifications; however, the following modifications were made: the frozen cell pellets were suspended in a 1 ml Kit-supplied SLX Mlus buffer containing 500 mg glass beads and ground at 65 Hz for 90 s on Tissue Lyser-24 (Jingxin, China). Cell fragment suspensions were immediately disposed of in accordance with instructions in the kit manual. Finally, the total DNA was obtained using an elution buffer of 50 μl.

### ITS1 amplification, quantification, and sequencing

The first internal transcribed spacer (ITS1) region of the fungus was amplified. Polymerase chain reaction (PCR) amplified 35 cycles of the target marker gene. Error-corrected 12 bp barcode primers were used for each sample to allow sample multiplexing. PCR products from all samples were quantified using PicoGreen dsDNA analysis and were aggregated at equimolar concentrations. Each library was submitted to Mega Biotech on the Illumina MiSeq PE300 platform. The raw reads have been deposited in the NCBI Sequence Read Archive (SRA) database, with accession number PRJNA718425.

### Sequence data analysis

FASTP (V0.20.0) was used to perform multiplex isolation and quality screening of the original ITS1 region sequencing reads (Chen et al., 2018) and using FLASH (V1.2.7) for merging. UPARSE (V7.1) was used to cluster the operational taxonomic units (OTU) of the sequences based on 97% similarity, and single sequences and chimera were removed. Each OTU representative sequence was classified and analyzed using the RDP Classifier (V2.2) and ITS database (e.g., Unite V8.0), with a confidence threshold of 0.7. To better convey the biological information of these samples, bar charts were used to visualize the average relative abundance of fungal communities at the phylum and genus levels.

### Statistical analysis

First, the Kruskal–Wallis H test and Wilcoxon rank-sum test were used to detect differences in the abundance of dominant fungi at the phylum and genus levels, respectively. This was conducted among different bamboo species in the same season and the same bamboo species in different seasons. We used the linear discriminant analysis (LDA) effect size (LEfSe; Segata et al., 2011) to identify the taxa that characterized the differences among three bamboo species (LDA score ≥ 3.5). The Sobs index and Shannon index were calculated for each sample using Motherur (V1.30.1) to estimate the species abundance and diversity of different bamboo species in spring and autumn, and the Wilcoxon rank-sum test was used to determine the significant level. Then, a Principlal Coordinate Analysis (PCoA) distance matrix based on the (un)weighted UniFrac method was used to evaluate the fungal community structure. Permutational MANOVA (PERMANOVA) was used to analyze the effects of different bamboo species and seasons on the fungal community structure based on the Bray–Curtis distance matrix.

Secondly, using the Spearman correlation analysis method, analysis of the relative abundance of fungal community and environmental factors (Supplementary Table S1). To investigate the spatial correlation between environmental factors and fungal communities, the Mantel test (based on the OUT level) was used to analyze the spatial correlation between the distance matrix of environmental factors and the UniFrac distance matrix of fungi. The canonical correspondence analysis (CCA) was used to determine samples, environmental factors, and the relationship between the fungi flora. The relationship between Alpha (the Sobs index and Shannon index) and Beta diversity (Bray-Curtis distance) and environmental factors was analyzed by the linear regression method.

Finally, the FUNGuild (Fungi Functional Guild) prediction method was used to predict the functional composition of fungi (Nguyen et al., 2016).

### Metagenome sequencing and analysis

Whole-genome shotgun sequencing using the Illumina sequencing HiSeq platform. The raw sequencing data were filtered for low-quality and host genomic bases using MetaGene (Sun et al., 2018). Through the CD-HIT (V4.6.1) for the sample prediction of gene sequence clustering (parameters: 90% identity and 90% coverage), non-redundant gene sets were constructed. SOAPaligner (V2.21) was used (Li et al., 2008; Fu et al., 2012), mapped the quality read data of each sample to the non-redundant gene set (parameter: 95% identity), and counted the abundance of genes in the sample. Non-redundant genes sets were compared with the NCBI NR database using DIAMOND (V0.8.35; Buchfink et al., 2015; parameter: Blastp. *E*-value ≤ 1E-5) to obtain the taxonomic annotation from the NR library. DIAMOND (V0.8.35) was used to map the non-redundant gene sets to Kyoto Encyclopedia of Genes and Genomes database to annotate the KEGG function. Non-redundant gene sets were compared with the CAZy database using HMMER (V3.1b2; parameter: Hmmscan. *E*-value ≤ 1E-5) to annotate carbohydrate-active enzyme. DIAMOND (V0.8.35) was used to map the non-redundant gene sets into CARD to obtain the annotation results for fungal antibiotic resistance genes (ARGs) in AS phyllospheres.

LEfSe was used to determine the relative abundance characteristics of the KEGG Level 2 (LDA score ≥ 2), CAZyme (LDA score ≥ 3), and CARD databases (LDA score ≥ 3.5) of the AS phyllosphere fungal community between spring and autumn (Segata et al., 2011).

### Co-occurrence network analysis

Using the OTU data of bacteria and fungi obtained by sequencing, OTUs with relative abundances >0.1% were selected. Network analyses were performed in R. Gephi (Version 0.9.2) used to construct microbial co-occurrence networks among AS, YL, and FF in spring and autumn. Antibiotic resistance genes with relative abundances >0.1% were selected, and network analyses were also performed in R. Gephi (Version 0.9.2) used to construct microbial co-occurrence network in AS. If the Spearman’s correlation coefficient |*ρ*| is not <0.8, and *p* values are no more than 0.01, suggests that a correlation between two items was considered statistically robust (Quintela-Baluja et al., 2019).

## Results

### Phyllosphere fungal alpha and beta diversity

A maximum of 19 samples were collected from each bamboo species in each study season (Supplementary Table S2). A total of 101 bamboo samples and 6,749,062 reads were obtained. The average target ITS1 reads of each sample were 66,822 ± 6,323. A total of 8,769 OTUs were obtained by clustering 97% similarity, among which 3,278 OTUs were shared among the three bamboo species (Supplementary Figure S1A). The fungal OTUs in the three bamboo species in autumn were significantly higher than those in spring (Supplementary Figures S1B, S2). Among the three bamboo species, FF had the highest number of OTUs in spring and autumn (Supplementary Figure S1A).

**Figure 2:**
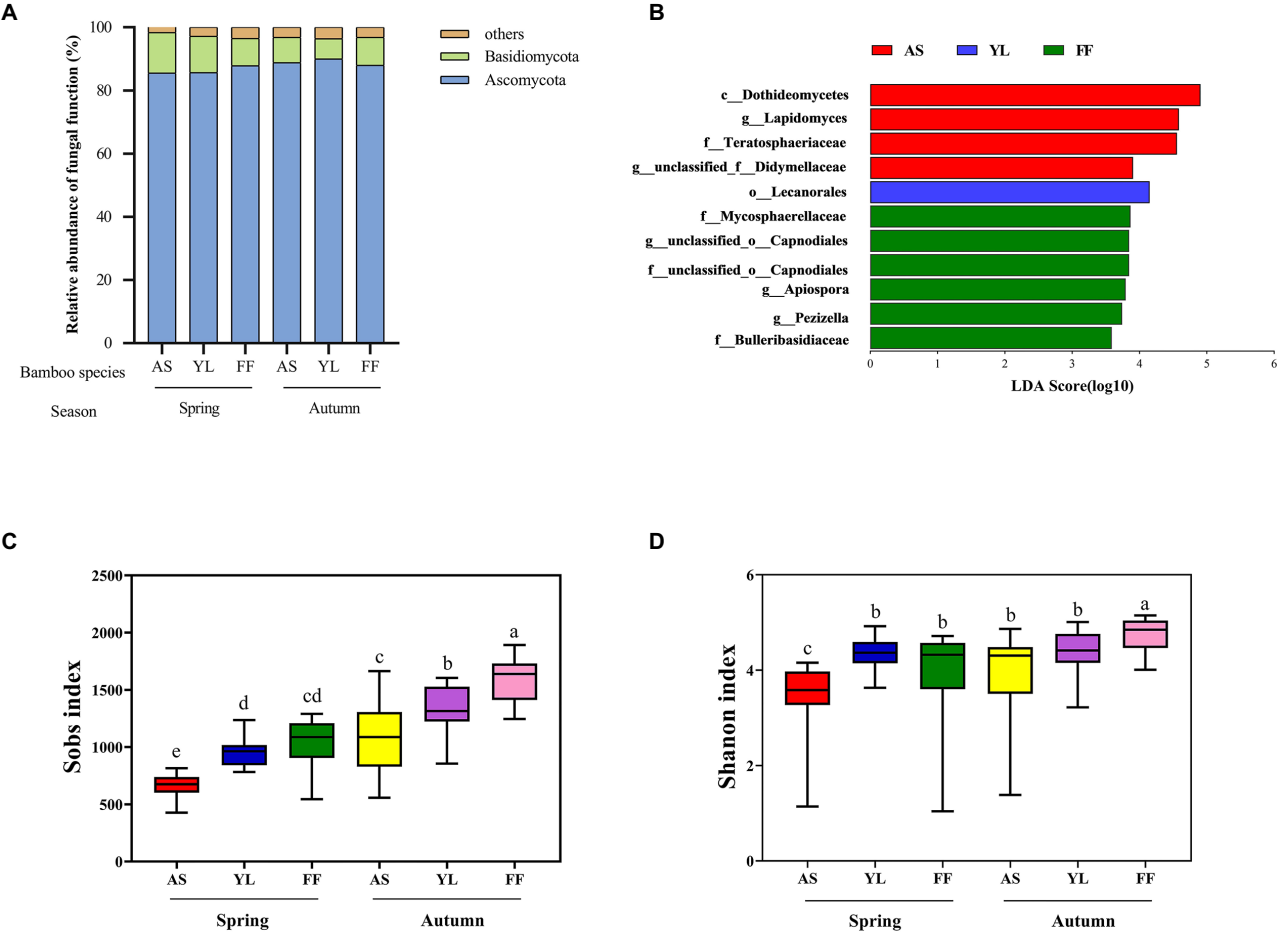
Taxonomic profiles of fungal communities among AS, YL, and FF phyllosphere. **(A)** Relative abundance of different phyllosphere fungi phyla among AS, YL, and FF in spring and autumn. **(B)** LEfSe analysis of phyllosphere fungi among AS, YL, and FF (LDA score ≥ 3.5). The Sobs index **(C)** and Shannon index **(D)** of AS, YL, and FF phyllosphere fungi in spring and autumn.

Taxonomic analysis classified fungal OTUs into 9 phyla and 1,053 genera. Most of the phyllosphere fungal community belonged to the phyla Ascomycota in all samples (Figure 2A). The rest comprised Basidiomycota and other phyla. The relative abundance of the phyla Ascomycota and Basidiomycota were not significantly different among the three bamboo species (Figure 2A, Supplementary Figures S4A,B; Supplementary Table S3). The relative abundance of the phylum Basidiomycota in YL phyllosphere was higher in spring than in autumn, and the relative abundance of the phylum Ascomycota in YL phyllosphere was lower in spring than in autumn (Figure 2A; Supplementary Table S3).

At the genus level, unclassified_p__Ascomycota, *Trichomerium*, unclassified_c__Sordariomycetes, unclassified_o__Helotiales, unclassified_o__Pleosporales, and *Lapidomyces* were the dominant phyllosphere fungi (Supplementary Figure S3). In spring, the relative abundance of unclassifed_p__Ascomycota was significantly higher in the YL phyllosphere (Supplementary Figure S4C; Supplementary Table S3). The relative abundance of *Trichomerium* was significantly lower in the AS phyllosphere, but a contrasting pattern was observed for *Lapidomyces* (Supplementary Figure S4C; Supplementary Table S3). In autumn, the relative abundance of unclassified_p__Ascomycota and unclassified_o__Helotiales were significantly higher in the YL phyllosphere (Supplementary Figure S4D; Supplementary Table S3). The highest relative abundance of unclassified_o__Pleosporales existed in the AS phyllosphere, but a contrasting pattern was observed for unclassified_c__Sordariomycetes (Supplementary Figure S4D; Supplementary Table S3). For all three bamboo species, the relative abundance of unclassified_c__Sordariomycetes in autumn was significantly lower than that in spring (Supplementary Table S3).

LEfSe analysis revealed that the LDA scores of microbial taxa differed significantly among AS, YL, and FF (Figure 2B). Four fungi groups (c__Dothideomycetes, g__Lapidomyces, f__Teratosphaeriaceae, and g__unclassified_f__Didymellaceae) in the AS phyllosphere were significantly enriched in comparison to the other two bamboo species. In YL phyllosphere, only o__Lecanorales was significantly enriched in comparison to the other two bamboo species. In FF phyllosphere, six groups of fungi (including f__Mycosphaerellaceae, g__unclassified_o__Capnodiales, f__unclassified_o__Capnodiales, g__Apiospora, g__Pezizella, and f__Bulleribasidiaceae) were significantly enriched in comparison to the other two bamboo species (Figure 2B).

The Sobs index and Shannon index were found to decrease from autumn to spring in all bamboo species (Figures 2C,D; Supplementary Table S4). AS phyllosphere fungal community had significantly lowest the Sobs index and Shannon index in spring (Figures 2C,D; Supplementary Table S4). In autumn, the Sobs index and Shannon index in FF phyllosphere were significantly higher than those of AS and YL (Figures 2C,D; Supplementary Table S4).

**Figure 3:**
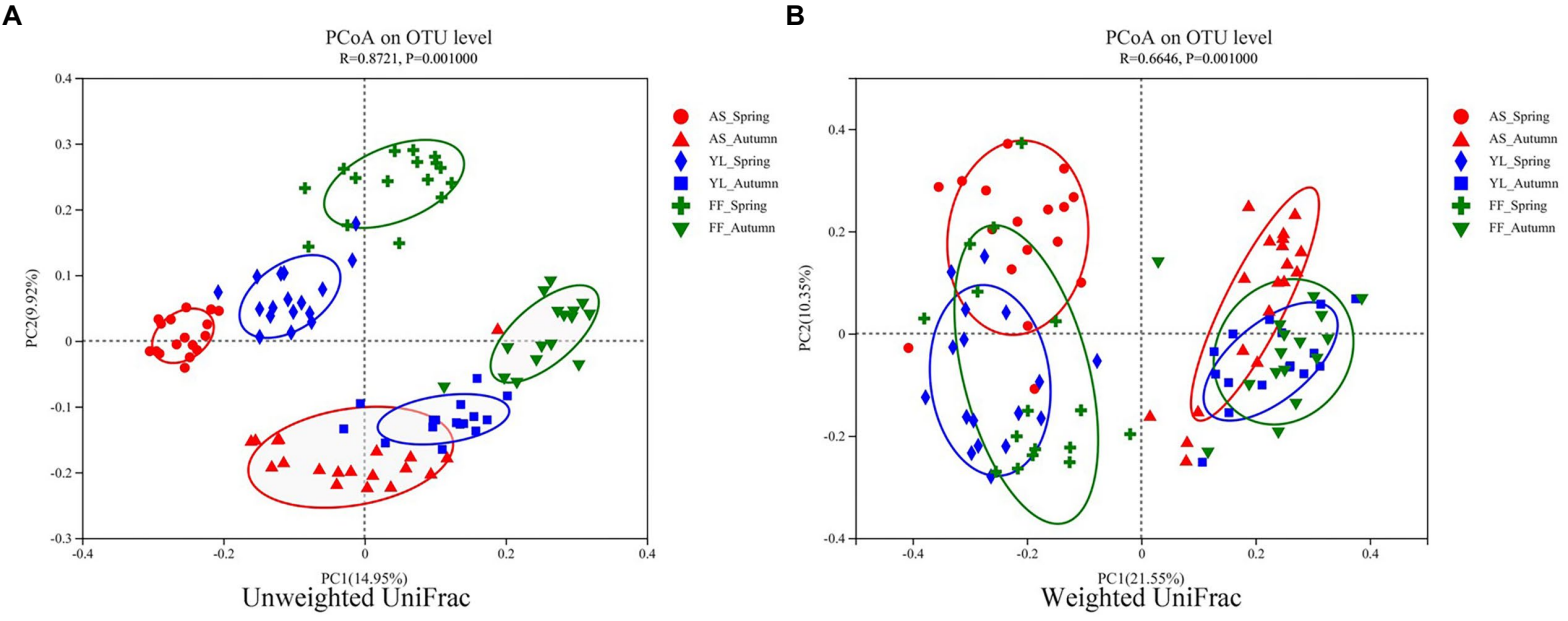
PCoA of phyllosphere fungal communities based on the (un)weighted UniFrac distance matrixes **(A,B)** among AS, YL, and FF in spring and autumn.

The PCoA results showed that the samples were clustered by season and species (Figure 3). Based on weighted UniFrac, three bamboo species clustered together in the same season, FF and YL clustered closer together. Based on unweighted UniFrac, the three bamboo species are easier to distinguish than the weighted UniFrac (Figure 3). Further, the PERMANOVA based on the Bray-Curtis distance matrix showed that there were significant differences in the phyllosphere fungal community structure between the three bamboo species in spring and autumn (Table 1).

**Table 1:** PERMANOVA exploring the effects of three bamboo species and two seasons based on Bray–Curtis distance matrixes.

Grouping factors	Sums of Sqs	Mean Sqs	*F* model	*R* ^2^	Value of *p*
AS (Spring:Autumn)	2.298	2.298	11.947	0.260	0.001
YL (Spring:Autumn)	2.017	2.017	10.813	0.258	0.001
FF (Spring:Autumn)	1.611	1.611	7.132	0.192	0.001
Spring:Autumn	12.109	2.422	12.047	0.388	0.001
Autumn (AS:YL:FF)	3.568	1.784	8.845	0.269	0.001
Spring (AS:YL:FF)	4.843	2.422	12.087	0.340	0.001

### Ecological factors influencing bamboo phyllosphere fungal communities

**Figure 4:**
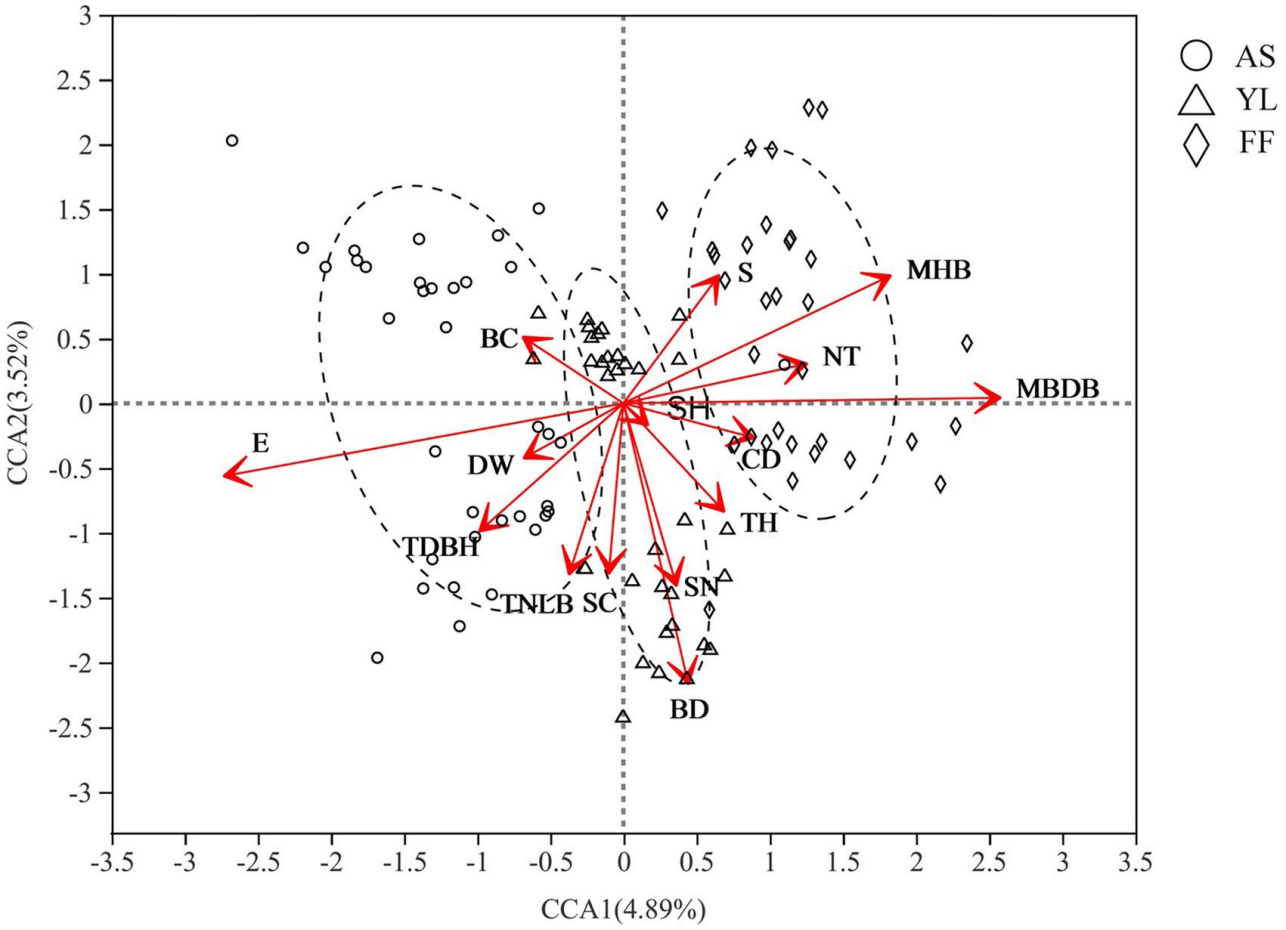
CCA analysis of environmental factors and phyllosphere fungal community among AS, YL, and FF. E, elevation; DW, water source distance; TDBH, trees diameter at breast height; TNLB, total number of live bamboo; SC, shrub coverage; BD, bamboo deaths; SN, shrubs numbers; TH, trees height; SH, Shrub height; CD, canopy density; MBDB, mean base diameter of bamboo; NT, number of trees; MHB, mean height of bamboo; S, Slope; BC, bamboo coverage.

Mantel analysis results showed that elevation had the greatest effect on the phyllosphere fungal community (*R* = 0.617, *p* = 0.001; Supplementary Table S5). Furthermore, CCA showed that elevation (E), mean base diameter of bamboo (MBDB), mean height of bamboo (MHB), bamboo deaths (BD), tree diameter at breast height (TDBH), shrub number (SN), total number of live bamboo (TNLB), number of trees (NT), shrub coverage (SC), slope (S), tree height (TH), canopy density (CD), and water source distance (DW) also influenced the fungal community (Figure 4; Supplementary Table S6).

**Figure 5:**
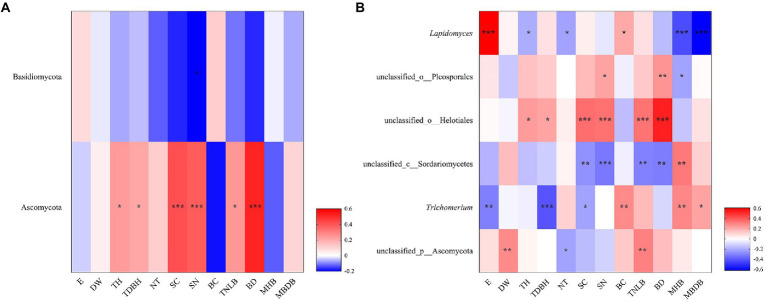
Correlation heatmap showed the relative abundance of fungi phylum **(A)** and genus **(B)** in relation to environmental factors. R is shown in different colors, and the legend on the right is the color range of different R values. E, elevation; DW, water source distance; TDBH, trees diameter at breast height; TNLB, total number of live bamboo; SC, shrub coverage; BD, bamboo deaths; SN, shrubs numbers; TH, trees height; SH, Shrub height; CD, canopy density; MBDB, mean base diameter of bamboo; NT, number of trees; MHB, mean height of bamboo; S, Slope; BC, bamboo coverage. (^*^0.01 < *p* < 0.05, ^**^0.001 < *p* < 0.01, ^***^p < 0.001).

The Spearman’s correlation heatmap showed that Basidiomycota abundance was significantly negatively correlated to shrub number (SN; Figure 5A). Ascomycota abundance was significantly positively correlated to tree height (TH), tree diameter at breast height (TDBH), shrub coverage (SC), shrub number (SN), total number of live bamboo (TNLB), and bamboo deaths (BD; Figure 5A). At the genus level, *Lapidomyces* exhibited significantly positive correlations with elevation (E) and bamboo coverage (BC), but significantly negative correlations with tree height (TH), number of trees (NT), mean height of bamboo (MHB), and mean base diameter of bamboo (MBDB; Figure 5B). unclassified_o__Helotiales and unclassified_o__Pleosporales were both positively correlated with tree height (TH), tree diameter at breast height (TDBH), shrub coverage (SC), shrub number (SN), total number of live bamboo (TNLB), and bamboo deaths (BD). unclassified_c__Sordariomycetes exhibited significantly negative correlations with shrub coverage (SC), shrub number (SN), total number of live bamboo (TNLB), and bamboo deaths (BD), but significantly positive correlations with mean height of bamboo (MHB). *Trichomerium* exhibited significant positive correlations with bamboo coverage (BC), mean height of bamboo (MHB), and mean base diameter of bamboo (MBDB), but negative correlations with elevation (E), tree diameter at breast height (TDBH), and shrub coverage (SC). unclassified_p__Ascomycota was significantly positively correlated to water source distance (DW) and total number of live bamboo (TNLB), but negatively correlated to number of trees (NT; Figure 5B).

Further linear regression revealed that elevation had the highest effect for the Bray–Curtis distance, but the highest ecological influence factor on the Sobs index and Shannon index was mean base diameter of bamboo (Supplementary Table S7).

### Co-occurrence patterns of fungal interactions with bacteria community

**Figure 6:**
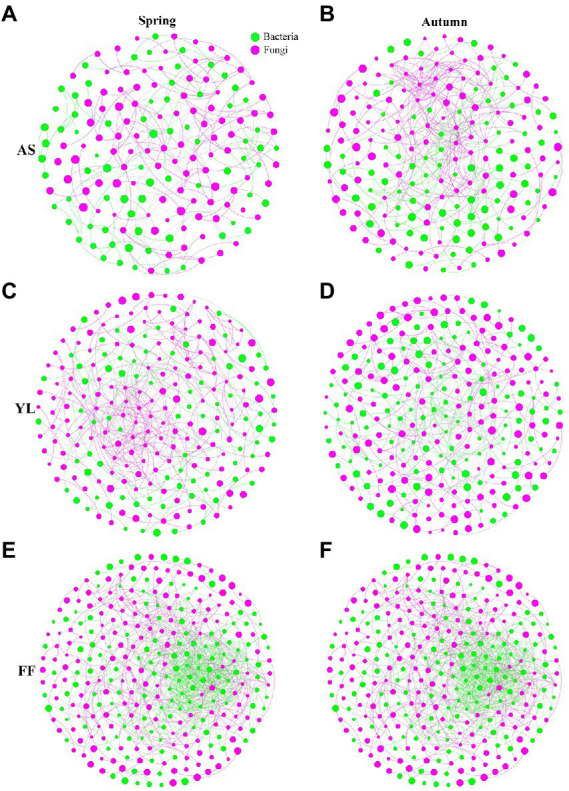
Co-occurrence network analysis showing the fungi interactions with bacteria among AS, YL, and FF phyllosphere in spring and autumn **(A–F)**.

The results of co-occurrence network analysis showed that the network complexity, node connectivity, and average degree of the *F. ferax* microbial community were higher than those of *A. spanostachya* and *Y. lineolate* (Figure 6; Supplementary Table S8). In autumn, *A. spanostachya* communities employed a much more complex network than that in spring (Figures 5A,B), which was reflected in the node and edge number, as well as average degree, which was higher in autumn than in spring (Supplementary Table S8). However, the network complexity of *F. ferax and Y. lineolate* had no obvious differences in the two seasons (Figures 6C–F). *F. Ferax* microbial networks were mainly reflected in the interactions within the bacterial domain. *A. spanostach* and *Y. lineolate* microbial networks were mainly reflected in the interactions within the fungal domain (Figure 6; Supplementary Table S8).

### Function of the fungi (e.g., KEGG, CAZy, and ARGs)

The functional classification of fungi was performed based on the Fungi Functional Guild (Nguyen et al., 2016). Three ecological guilds – pathotroph, saprotroph, and symbiotroph – for ~40% of all fungal OTUs were detected (Supplementary Figure S5).

**Figure 7:**
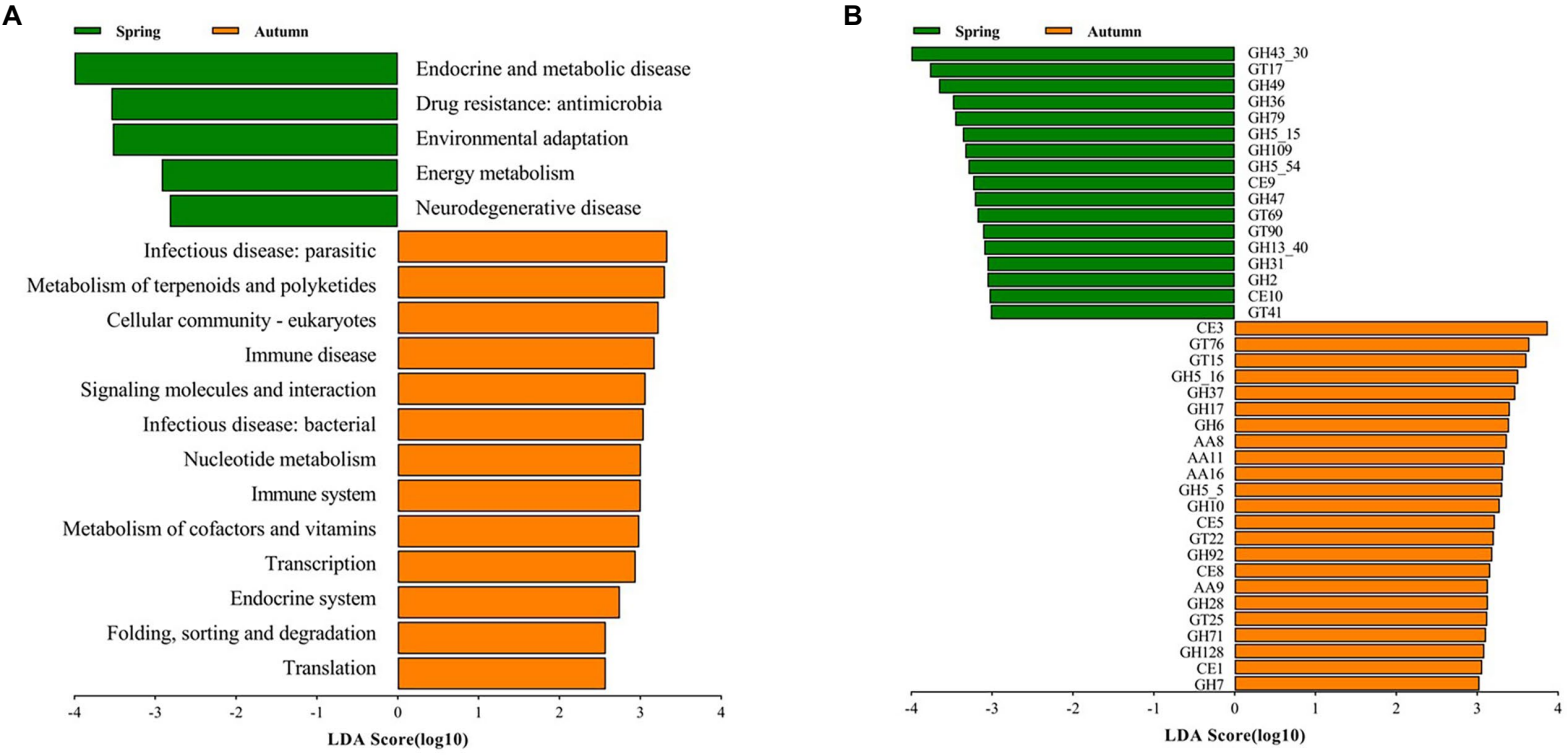
LEfSe analysis of KEGG level 2 **(A)** (LDA score ≥ 2) and CAZy **(B)** (LDA score ≥ 3) of AS phyllosphere fungal community between spring and autumn. GH, glycoside hydrolases; GT, glycosyl transferases; CE, carbohydrate esterases; AA, auxiliary activities.

In order to further explore the variation of functional potential in AS phyllosphere, we screened 8 samples (spring: 4 samples; autumn: 4 samples) for whole-genome shotgun sequencing. A total of 3.91 million contigs and 2.47 Gb of assembly sequence were obtained, and the average contig N50 was 655 bp (Supplementary Table S9). The metagenomic analysis confirmed 402 KOs, including 46 KEGG Level 2 categories in fungi. In the KEGG level 2 categories, the relative abundance of global and overview maps (20%) was the highest in *A. spanostachya*, followed by neurodegenerative disease (7%), signal transduction (6%), and carbohydrate metabolism (4%; Supplementary Figure S6A). In spring, AS phyllosphere fungi showed high abundances in KEGG Level 2 categories of neurodegenerative disease, energy metabolism, environmental adaptation, and drug resistance: antimicrobial, endocrine, and metabolic disease (Figure 7A), and the functions of AS phyllosphere fungi are primarily a consequence of the fungi of the *Beauveria* and *Cladosporium* genus (Supplementary Table S10). In contrast, in autumn, translation; folding; sorting and degradation; endocrine systems; transcription; metabolism of cofactors and vitamins; immune systems; nucleotide metabolism; infectious diseases: bacterial, signaling molecules, and interactions; immune disease; cellular community-eukaryotes; metabolism of terpenoids and polyketides; and infectious diseases: parasitic, exhibited higher abundance (Figure 7A), and the functions of AS phyllosphere fungi are mainly a consequence of fungi of the *Beauveria* and *Lachnellula* genus (Supplementary Table S10).

The relative abundance of CAZy in glycoside hydrolases (43%) was the highest in AS phyllosphere fungi which contained some cellulases (GH5, GH6, GH7, GH28, etc.), followed by glycosyl transferases (27%), auxiliary activities (16%), and carbohydrate esterases (11%; Supplementary Figure S6B; Supplementary Table S10). According to the LEfse results for the CAZyme, three families had significantly higher relative abundances in spring, including glycoside hydrolases (GHs), glycosyl transferases (GTs), and carbohydrate esterases (CEs; Figure 7B), and the functions of AS phyllosphere fungi are mainly a consequence of fungi of the Chaetothyriales and Hypocreales order (Supplementary Table S11). In autumn, four families had significantly higher relative abundances: GHs, CEs, GTs, and AAs, and the functions of AS phyllosphere fungi are mainly a consequence of fungi of the Chaetothyriales and Pleosporales orders (Supplementary Table S10). Interestingly, GHs had the highest differences between spring and autumn (Figure 7B).

**Figure 8:**
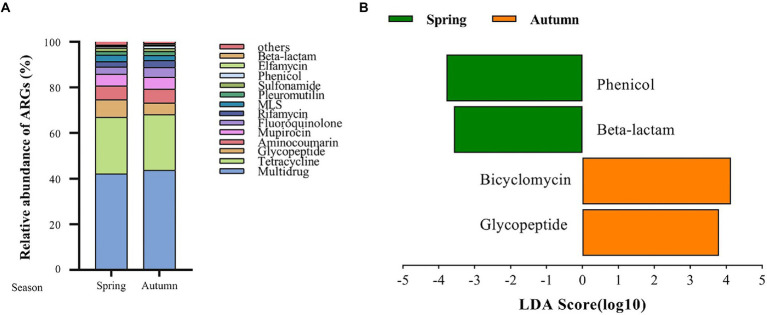
Fungal antibiotic resistance genes (ARGs) in AS phyllosphere. Relative abundance of fungal antibiotic resistance genes (ARGs) in AS phyllosphere between spring and autumn **(A)**. LEfSe analysis of fungal antibiotic resistance genes (ARGs) in AS phyllosphere between spring and autumn **(B)**. (LDA score ≥ 3.5).

**Figure 9:**
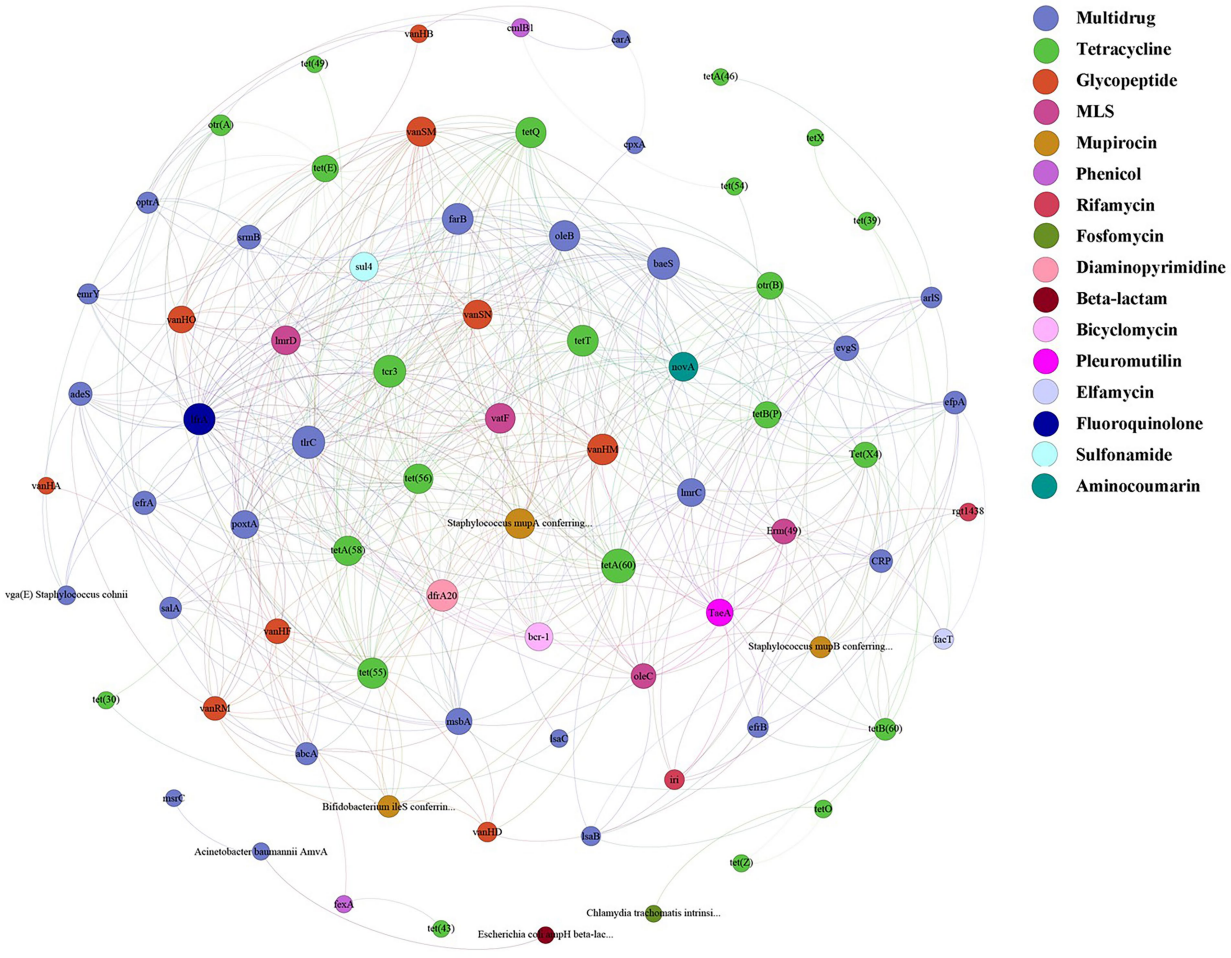
ARGs co-occurrence network of fungal resistance genes in AS phyllosphere. Nodes represent genes and are colored to indicate antimicrobial classes of ARGs.

A total of 125 antibiotic resistance genes were detected in eight bamboo samples from the AS phyllosphere fungi based on metagenomic sequencing (Supplementary Table S11). At the genus level, g__unclassified_o__Helotiales, g__Epicoccum, g__Mortierella, and g__Paraphaeosphaeria had multidrug resistance genes; g__unclassified_k__Fungi had multidrug and tetracycline resistance genes; g__Periconia had aminocoumarin resistance genes; g__Lachnellula had multidrug, tetracycline, and rifamycin resistance genes; g__Beauveria had multidrug, tetracycline, sulfonamide, fluoroquinolone, pleuromutilin, and beta-lactam resistance genes (Supplementary Table S11). Multidrug (42%) and tetracycline (24%) resistance genes were the most common types of ARGs in all samples (Figure 8A). The abundance of multidrug, tetracycline, and glycopeptide resistance genes was high and closely correlated with other ARGs (Figure 9). Beta-lactam resistance gene had only one edge to multidrug resistance genes (Figure 9). The remaining ARGs were glycopeptide, aminocoumarin, mupirocin, fluoroquinolone, rifamycin, etc. According to the LEfse results of the CARD, in spring, AS fungi displayed higher abundances of beta-lactam and phenicol resistance genes than in autumn; however, glycopeptide and bicyclomycin resistance genes exhibited higher abundance in autumn (Figure 8B).

## Discussion

In our study, Ascomycota and Basidiomycota were found to be the dominant phyla in AS, YL, and FF phyllosphere fungi microflora, which is similar to several previous studies (Helander et al., 2013; Xu et al., 2014; Jin et al., 2020). The fungal OTUs richness and diversity (e.g., Sobs index and Shannon index) of the three bamboo species in spring were significantly lower than those in autumn. This result matches that found by Zheng (2011), who determined that the diversity of *Pinus tabulaeformis* phyllosphere microbial communities exhibited the greatest diversity in autumn, followed by summer and spring. Further, Thompson et al. (1993) also found that the diversity of phyllosphere fungal communities of *Beta vulgaris* in spring was lower than that in autumn. The season is an important factor affecting phyllosphere microorganisms, whose diversity is higher in autumn than in spring because of higher temperature and humidity in autumn (Laforest et al., 2016).

The season is also a stronger driver of the phyllosphere fungal community structure than the host bamboo species based on the (un)weighted UniFrac distance matrix (Figure 3). However, this result differs from that of Laforest et al. (2016), who found that host species are a more important factor than the site or time in terms of determining the community of temperate tree phyllospheres.

The Mantel test found that elevation, water source distance, tree height, etc. significantly affected the phyllosphere fungal community, and effect of elevation is the most obvious (Supplementary Table S5), which is similar to the result of Long et al. (2021), who found that elevation, water source distance, etc. significantly impact the phyllosphere bacterial community. Similarly, Cordier et al. (2012) found that elevation affects the relative abundance and community composition of *Fagus sylvatica* fungal communities. Furthermore, CCA found that slope significantly affected the phyllosphere fungal community. Similarly, Bari et al. (2019) explored the effects of elevation, the field slope, site direction, and fungal spore fruit position height on trees, finding that the field slope was the main factor influencing the fungal spores abundance.

To explore the effect of the micro-ecological environment (biotic factor) on the bamboo phyllosphere fungal community, a co-occurrence network analysis was performed, which showed that FF had higher network complexity than AS and YL. This may be related to the higher Sobs and Shannon indices in FF phyllosphere microbial communities, because the higher Sobs and Shannon indices would increase the possibility of interactions between different microbial communities (Wang B. et al., 2022). This result was similar to those of Wang B. et al. (2022), who found that the high alpha diversity of microbial community in a given group provides additional potential interactions between groups. AS had higher network complexity in autumn than in spring. However, networks of FF and YL slightly differed in spring and autumn (Figure 6). It is possible that AS occurs at high altitudes and has more distinct climatic environments, in terms of both temperature and light. There are complex links between bacteria and fungi, bacteria and bacteria, and fungi and fungi in AS, YL, and FF. Bacteria and fungi usually have symbiotic relationships, with both positive and negative effects. For example, some bacteria stimulate mycorrhizal formation, and fungi and bacteria grow faster when they are present together (Bengtsson, 1992; Founoune et al., 2002; Mille-Lindblom et al., 2006); bacterial volatiles inhibit the growth of fungal spores, and fungal volatiles inhibit not only the growth of bacteria, but also the growth of other fungi (Schmidt et al., 2015).

In this study, the functions of AS phyllosphere fungi were mainly dominated by *Beauveria* (Supplementary Table S10), a genus of entomogenic fungi that plays an important role in pest control and has no pathogenic effect on humans and animals (Mascarin and Jaronski, 2016). *Beauveria* not only protects giant panda’s food resources but can also kill giant panda’s ectoparasites. Carbohydrate metabolism and GH enzymes are mainly a consequence of Eurotiomycetes and Dothideomycetes (Supplementary Table S10). It has been demonstrated that Eurotiomycetes and Dothideomycetes consume lignin when it is the only carbon source (Ferrari et al., 2021). The majority of vitamins come from an animal’s diet. Both the giant panda and red panda forage on AS as a staple food in Liziping NR (Wei et al., 1999). The abundant metabolism of cofactors and vitamins may be beneficial to the physical and mental health of giant pandas and red pandas (Badawy, 2014; Rudzki et al., 2021). Further, the abundant energy metabolism and environmental adaptation in spring indicated that spring was not a suitable season for phyllosphere fungi (Laforest et al., 2016). More abundant translation, folding, sorting and degradation, transcription, nucleotide metabolism, signaling molecules and interactions, and cellular community-eukaryotes indicated fungal cell viability and cell proliferation in autumn.

The LEfse results of the CAZyme show that glycoside hydrolases (GHs) exhibit the main differences, which supports the fact that phyllosphere microorganisms rely primarily on carbohydrate metabolism for nutrients (Kawaguchi et al., 2011; Ryffel et al., 2016). GHs, which can decompose complex carbohydrates, are a highly diverse group of key enzymes related to gut microbiota and their metabolic function (Lee et al., 2013). Zhu et al. (2011) found that the gut microbiome of giant pandas contains a small number of cellulases, and giant pandas and red pandas may obtain cellulose-digesting flora from phyllosphere microorganisms by eating bamboo leaves in large quantities (Jin et al., 2020).

In the AS phyllosphere, multidrug and tetracycline resistance genes were the most dominant ARGs. ARG co-occurrence network analysis showed that beta-lactam resistance genes were rarely correlated with other ARGs (Figure 9), reducing the possibility of co-selection of other antimicrobials (Yang et al., 2022). Many phyllosphere fungi have multiple resistance genes, proving that phyllospheres have become reservoirs for resistance genes (Sun et al., 2021). Multidrug resistance genes have been shown to be the most abundant ARGs in some vegetables’ phyllosphere (Blau et al., 2018; Yin et al., 2022). Multidrug resistance to many different classes of antibiotics, which impedes antibiotic treatment, is a huge risk to human life (Zhu et al., 2013; Ouyang et al., 2015), and is also a potential threat to giant panda health. Several recent studies have shown that diverse ARGs exist in the gut of wild and captive giant pandas, and multidrug resistance genes were the dominant type of ARGs in the giant panda gut (Guo et al., 2019; Hu et al., 2020; Mustafa et al., 2021; Zhu et al., 2021). A survey based on ARGs of human gut microbiota in three countries (China, Denmark, and Spain) found that tetracycline resistance genes were the most abundant group of ARGs, followed by MLSs and beta_lactam resistance genes (Hu et al., 2013). Given the abundance and diversity of ARGs in the bamboo phyllosphere, the diet has proven to be the main driving factor for ARG variation in the gut microbiome of giant pandas (Zhu et al., 2021).

## Conclusion

Overall, our results indicated that the fungal community’s abundance, diversity, and community structure were mainly affected by the season, host species, and elevation. The season and host species are major factors affecting biological functions (KEGG and CAZyme), ARGs, and interactions between sympatric bacterial and fungal communities in bamboo phyllospheres. As the most important food resource for giant pandas in Liziping NR (Xiaoxiangling mountains; Hong et al., 2015; Sichuan Forestry Bureau, 2015; Xie and Hong, 2020), AS phyllosphere fungi contain many glycoside hydrolases (e.g., cellulases CH5, GH6, GH7, etc.), which may enter the giant pandas’ intestine through ingestion, helping them to digest cellulose and obtain nutrition. The functions of AS phyllosphere fungi were mainly dominated by *Beauveria*, which can be used for biological control of pests and diseases (Mascarin and Jaronski, 2016). Therefore, food microbes (bamboo phyllosphere microorganisms) may be an important technique for giant pandas’ gut microbes to degrade cellulose and also protect food resources of giant pandas (Zhu et al., 2011). Although various ARGs were found in AS phyllosphere fungi flora, these ARGs could naturally occur in bamboo phyllosphere fungi flora (D’Costa et al., 2011; Sun et al., 2021). Therefore, numerous comparative studies on the effects of human activities on bamboo phyllosphere microorganism ARGs should be carried out in giant panda habitats to predict the risk of antibiotics for wild pandas.

## Data availability statement

The datasets presented in this study can be found in online repositories. The names of the repository/repositories and accession number(s) can be found in the article/Supplementary material.

## Author contributions

LK: conceptualization, methodology, investigation, software, resources, formal analysis, and writing original draft. WL, QD, HZ, WW, JT, HH, YY, and JL: investigation, validation and data curation. ZZ: conceptualization, supervision, and writing–review and editing. MH: conceptualization, supervision, funding acquisition, writing–original draft, and review and editing. All authors contributed to the article and approved the submitted version.

## Funding

This study was supported by the National Natural Science Foundation of China (grant no. 31900337), The Second Tibetan Plateau Scientific Expedition (grant no. 2019QZKK05010502), and the Innovation Team Funds of China West Normal University (grant no. KCXTD2022-7).

## Conflict of interest

The authors declare that the research was conducted in the absence of any commercial or financial relationships that could be construed as a potential conflict of interest.

## Publisher’s note

All claims expressed in this article are solely those of the authors and do not necessarily represent those of their affiliated organizations, or those of the publisher, the editors and the reviewers. Any product that may be evaluated in this article, or claim that may be made by its manufacturer, is not guaranteed or endorsed by the publisher.
