# Supplementary material for: Giant pandas’ staple food bamboo phyllosphere fungal community and its influencing factors
Source: Front Microbiol. 2022 Sep 30;13:1009588. doi: 10.3389/fmicb.2022.1009588 (PMC9561849; doi:10.3389/fmicb.2022.1009588)
Supplement: Supplementary file 4 [file Data_Sheet_1.docx]

**Figure S1.** Venn diagram showing (non)overlap of phyllosphere fungi OTUs among three bamboo species (**A**) and two seasons (**B**).


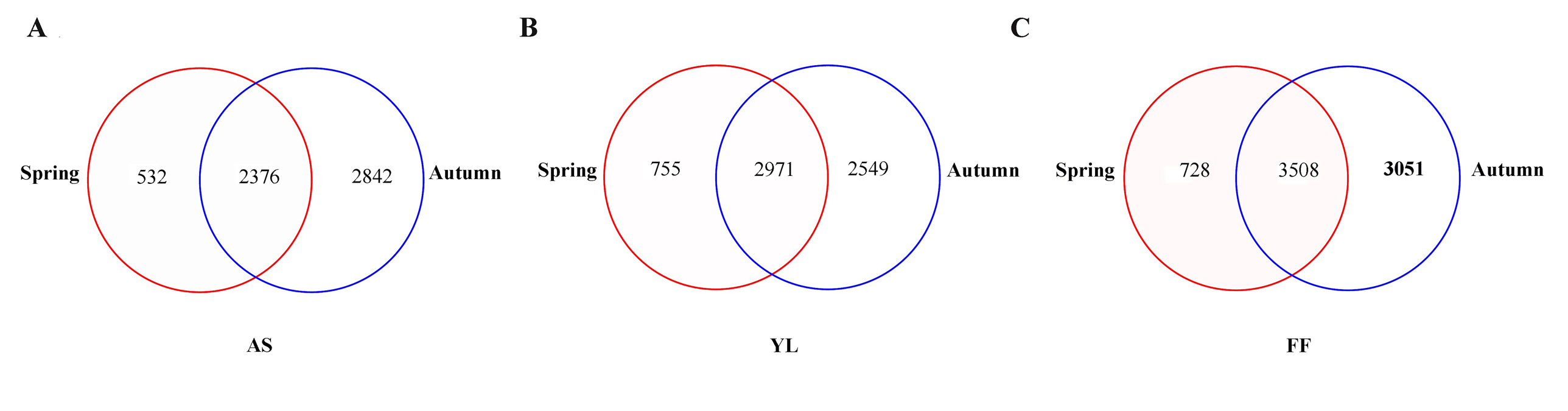


**Figure S2.** Venn diagram showing (non)overlap of phyllosphere fungi OTUs of AS (**A**), YL (**B**), and FF (**C**) between spring and autumn.


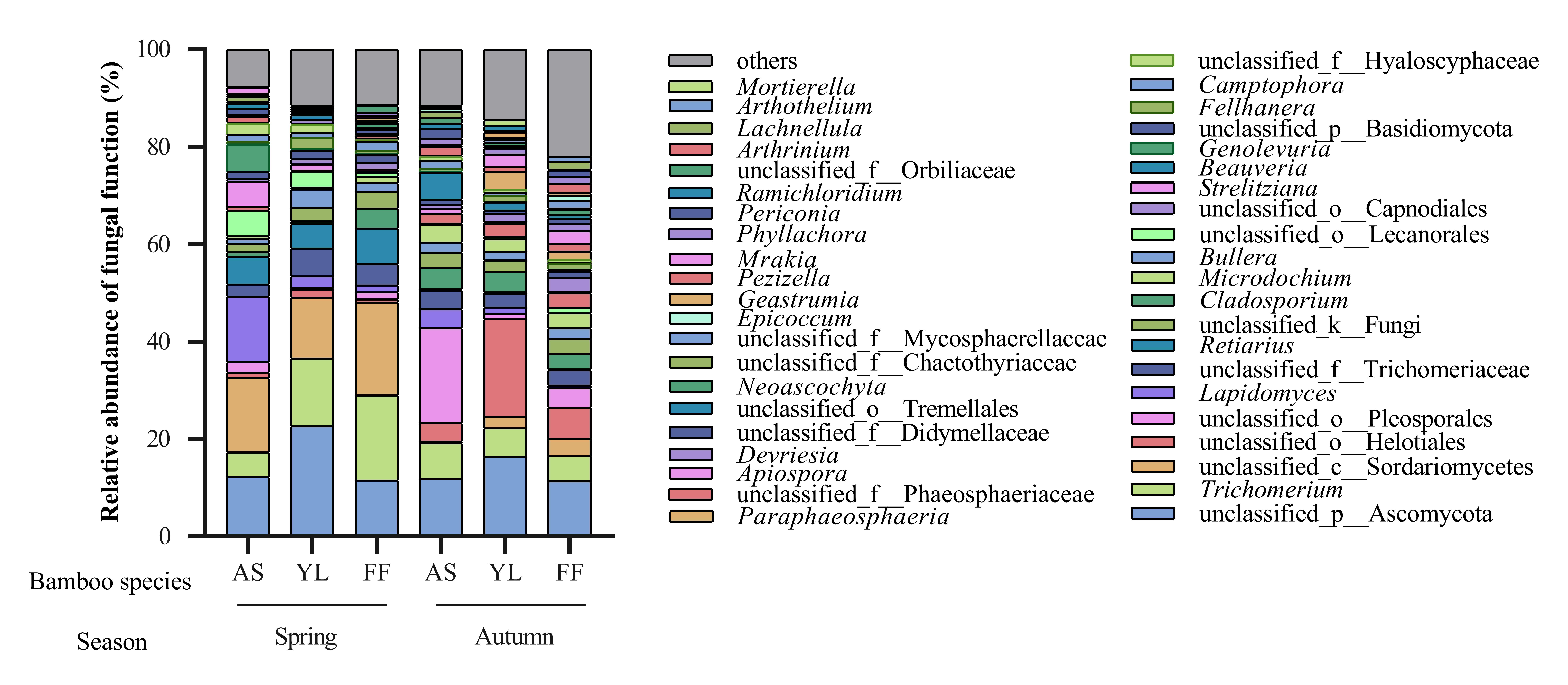


**Figure S3.** Relative abundance of different phyllosphere fungi genus among AS, YL, and FF in spring and autumn.





**Figure S4.** Comparing of relative abundance of phyllosphere dominant fungi phyla and genus among AS, YL, and FF throngh Kruskal-Wallis H test. Comparing of dominant phyla among AS, YL, and FF in spring (A) and autumn (B). Comparison of dominant genus among AS, YL, and FF in spring (C) and autumn (D).

**
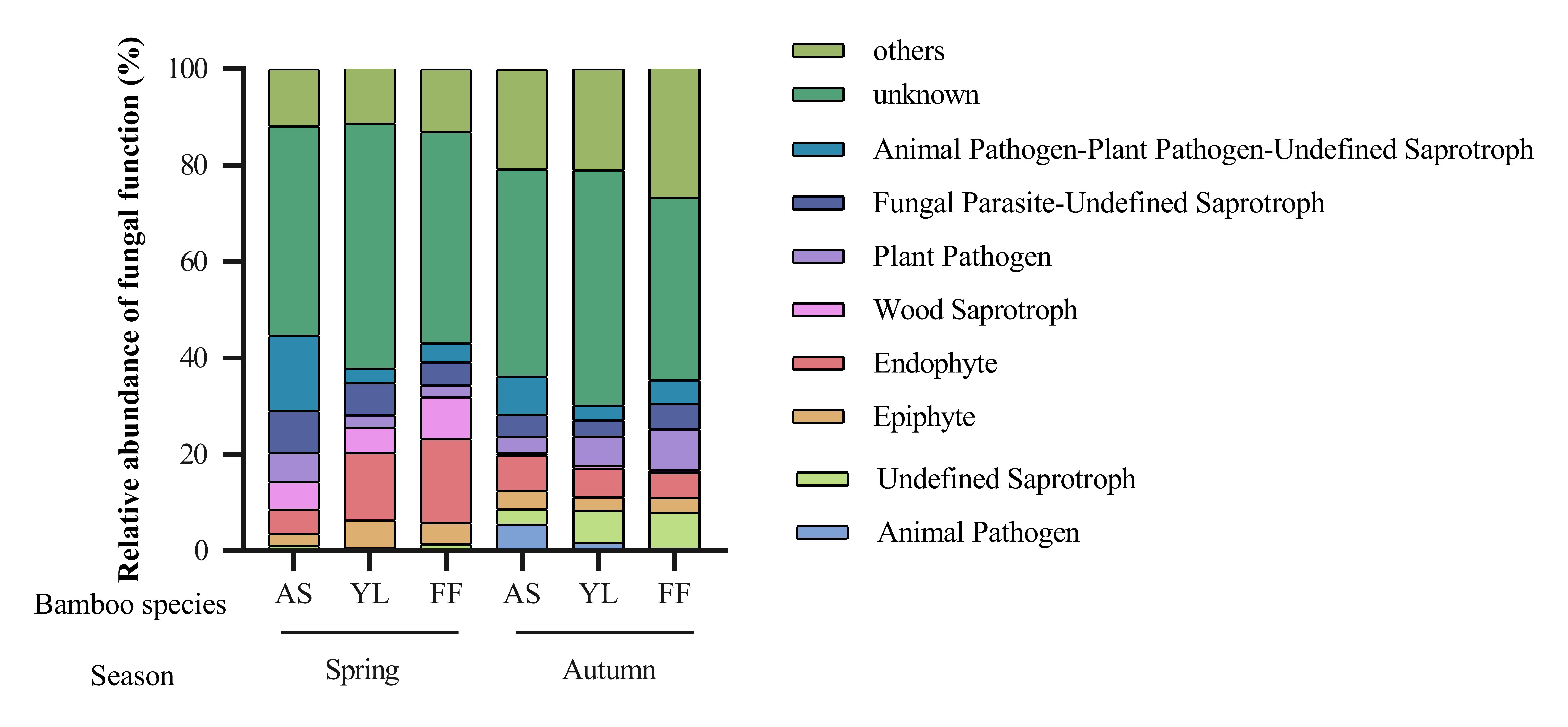
**

**Figure S5.** Variations in composition of fungal functional groups inferred by FUNGuild among three bamboo species in two seasons.


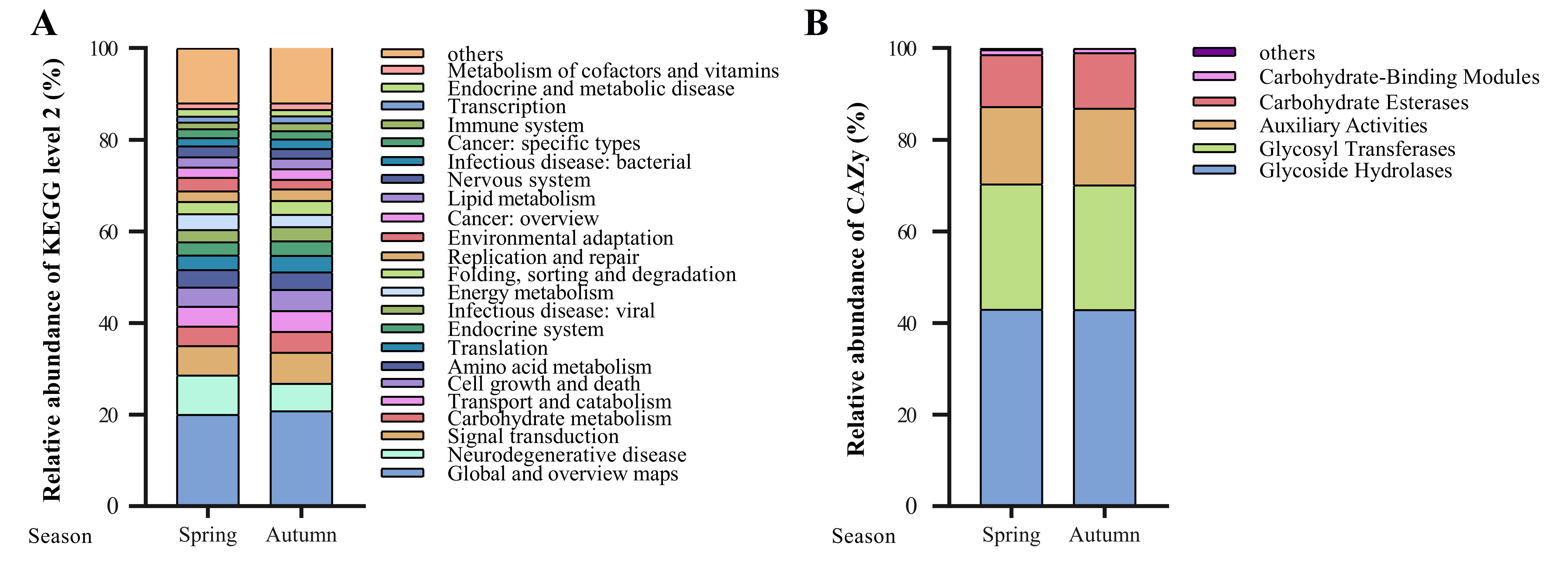


**Figure S6.** Relative abundance of KEGG level 2 (A) and CAZy (B) of AS phyllosphere fungal community between spring and autumn.
