# Supplementary material for: Giant pandas’ staple food bamboo phyllosphere fungal community and its influencing factors
Source: Front Microbiol. 2022 Sep 30;13:1009588. doi: 10.3389/fmicb.2022.1009588 (PMC9561849; doi:10.3389/fmicb.2022.1009588)
Supplement: Supplementary file 5 [file Data_Sheet_2.docx]

**Table S1.** Definition and measurement of each variable.

| Variables(Units) | Definition and Measurement |
| --- | --- |
| Elevation, E (m) | Elevation of central point of survey plot (20×20m^2^) |
| Slope, S (°) | Slope of central point of survey plot (20×20m^2^) |
| Water source distance, DW (m) | Distance from center point of survey plot (20×20m^2^) to visible or audible water flow |
| Tree height, TH (m) | Average height of trees over 5m in high in survey plot (20×20m^2^) |
| Tree diameter at breast height, TDBH (cm) | Mean TDBH of trees over 5m in high in survey plot (20×20m^2^) |
| Number of trees, NT | Number of trees over 5m in high in survey plot (20×20m^2^) |
| Canopy density, CD (%) | Canopy density of trees over 5m in high in survey plot (20×20m^2^) |
| Shrub height, SH (m) | Average height of shrubs less than 5m in high in survey plot (20×20m^2^) |
| Shrub coverage, SC (%) | Shrub coverage in survey plot (20×20m^2^) |
| Shrubs number, SN | Number of shrubs in survey plot (20×20m^2^) |
| Bamboo coverage, BC (%) | Average bamboo coverage (%) of 3 bamboo plot (1×1m^2^) |
| Total number of live bamboo, TNLB | Average number of live bamboo in 3 bamboo plot (1×1m^2^) |
| Bamboo deaths, BD | Average number of dead bamboo in 3 bamboo plot (1×1m^2^) |
| Annual number of bamboo | Average annual bamboo number of 3 bamboo plot (1×1m^2^) |
| Mean height of bamboo, MHB (cm) | The 3 bamboo plot (1×1m^2^) averaged annual bamboo height |
| Mean base diameter of bamboo, MBDB (mm) | The 3 bamboo plot (1×1m^2^) averaged annual bamboo base |
| Number of biennial bamboo | The 3 bamboo plot (1×1m^2^) mean biennial bamboo quantity |
| Biennial bamboo height (cm) | The average height of the 3 bamboo plot (1×1m^2^) was 2 years |
| Biennial bamboo base diameter (mm) | Three bamboo plot (1×1m^2^) averaged biennial growth of bamboo base meristem |
| Number of perennial bamboo | Average number of perennials of 3 bamboo plot (1×1m^2^) |
| Perennial bamboo height (cm) | Three bamboo plot (1×1m^2^) had average perennial bamboo height |
| Perennial bamboo base diameter (mm) | Three bamboo plot (1×1m^2^) averaged perennial bamboo base meristem |

**Table S2.** Number of samples and its effective sequences of AS, YL, and FF in spring and autumn.

| Season | Spring | | | Autumn | | |
| --- | --- | --- | --- | --- | --- | --- |
| Bamboo species | AS | YL | FF | AS | YL | FF |
| Number of samples | 17 | 17 | 16 | 19 | 16 | 16 |
| Number of effective sequences | 61,677±5,135 | 61,585±5,154 | 63,262±6,542 | 71,358±2,517 | 70,456±1,975 | 72,395±1,809 |

**Table S4.** The Sobs index and the Shannon index of phyllosphere fungal community among AS, YL, and FF in spring and autumn. (The data in the table is the mean ± standard deviation)

| **Diversity index** | **Autumn** | | | **Spring** | | |
| --- | --- | --- | --- | --- | --- | --- |
|  | **AS** | **YL** | **FF** | **AS** | **YL** | **FF** |
| Sobs index | 1105.32±292.63 | 1325.31±227.81 | 1589.56±191.98 | 670.82±104.77 | 956.41±125.79 | 1035.19±219.50 |
| Shannon index | 3.96±0.86 | 4.37±0.50 | 4.77±0.37 | 3.49±0.70 | 4.34±0.32 | 3.97±0.94 |

**Table S5.** Mantel tests exploring the association between environmental factors distance matrix and phyllosphere fungal UniFrac distance matrix.

| **Environmental factors** | **Fungal community structure** | |
| --- | --- | --- |
|  | ***R*** | ***P*** |
| E | 0.617 | **0.001** |
| S | 0.054 | 0.125 |
| DW | 0.169 | **0.001** |
| TH | 0.084 | **0.013** |
| TDBH | 0.141 | **0.002** |
| NT | 0.074 | **0.013** |
| CD | 0.063 | 0.090 |
| SH | 0.011 | 0.804 |
| SC | 0.070 | **0.025** |
| SN | 0.070 | **0.005** |
| BC | 0.126 | **0.001** |
| TNLB | 0.115 | **0.002** |
| BD | 0.099 | **0.009** |
| MHB | 0.285 | **0.001** |
| MBDB | 0.488 | **0.001** |

**Table S6.** CCA detecting the relationship between each environmental factor and phyllosphere fungal community in each sample based on OUT level.

| **Environmental factors** | **CCA1** | **CCA2** | **R^2^** | **p** |
| --- | --- | --- | --- | --- |
| E | -0.9807 | -0.1954 | 0.8455 | **0.001** |
| S | 0.6083 | 0.7937 | 0.1328 | **0.002** |
| DW | -0.8735 | -0.4868 | 0.0613 | **0.041** |
| TH | 0.6832 | -0.7302 | 0.1069 | **0.008** |
| TDBH | -0.7503 | -0.6611 | 0.1926 | **0.001** |
| NT | 0.9742 | 0.2255 | 0.1724 | **0.001** |
| CD | 0.9717 | -0.2362 | 0.085 | **0.009** |
| SH | 0.7539 | -0.657 | 0.0033 | 0.875 |
| SC | -0.1082 | -0.9941 | 0.1565 | **0.001** |
| SN | 0.2631 | -0.9648 | 0.1911 | **0.001** |
| BC | -0.8416 | 0.5401 | 0.0674 | **0.032** |
| TNLB | -0.3234 | -0.9463 | 0.1731 | **0.001** |
| BD | 0.2075 | -0.9782 | 0.4553 | **0.001** |
| MHB | 0.8989 | 0.4381 | 0.4547 | **0.001** |
| MBDB | 0.9994 | 0.0342 | 0.7191 | **0.001** |

**Table S7.** Linear Regression were conducted between environmental factors and Sobs index and Shannon index, and Bray-Curtis distance matrixes.

| **Ecology factors** | **Sobs** | | | **Shannon** | | | **Bray-Curtis distance** | | |
| --- | --- | --- | --- | --- | --- | --- | --- | --- | --- |
|  | **F** | **R²** | **p** | **F** | **R²** | **p** | **F** | **R²** | **p** |
| E | **40.374** | **0.290** | **0.000** | **14.543** | **0.128** | **0.000** | **120.113** | **0.548** | **0.000** |
| MBDB | **42.429** | **0.300** | **0.000** | **19.162** | **0.162** | **0.000** | **119.825** | **0.548** | **0.000** |
| BD | **13.567** | **0.121** | **0.000** | 3.360 | 0.033 | 0.070 | **11.943** | **0.108** | **0.001** |
| TH | **4.787** | **0.046** | **0.031** | 0.877 | 0.009 | 0.351 | **4.311** | **0.042** | **0.041** |
| MHB | **4.318** | **0.042** | **0.040** | **7.010** | **0.066** | **0.009** | **18.376** | **0.157** | **0.000** |
| NT | **4.036** | **0.039** | **0.047** | 0.424 | 0.004 | 0.517 | **12.339** | **0.111** | **0.001** |
| BC | 2.2345 | 0.022 | 0.138 | 0.870 | 0.009 | 0.353 | **4.407** | **0.043** | **0.038** |
| TNLB | 2.192 | 0.022 | 0.142 | **4.114** | **0.040** | **0.045** | 0.115 | 0.002 | 0.694 |
| TDBH | 2.107 | 0.021 | 0.150 | **6.146** | **0.059** | **0.015** | **4.734** | **0.046** | **0.032** |
| CD | 1.558 | 0.016 | 0.215 | 0.028 | 0.000 | 0.868 | **6.742** | **0.064** | **0.011** |
| SH | 1.198 | 0.012 | 0.277 | 0.066 | 0.001 | 0.797 | 0.271 | 0.003 | 0.604 |
| SN | 1.019 | 0.010 | 0.315 | 0.040 | 0.000 | 0.841 | **5.793** | **0.055** | **0.018** |
| S | 0.558 | 0.006 | 0.457 | 2.021 | 0.020 | 0.158 | 0.776 | 0.008 | 0.380 |
| DW | 0.178 | 0.001 | 0.674 | 0.567 | 0.006 | 0.453 | 1.901 | 0.019 | 0.171 |
| SC | 0.014 | 0.000 | 0.906 | 3.376 | 0.033 | 0.069 | 0.694 | 0.007 | 0.407 |

**Table S8.** Parameters of microbial co-occurrence network among AS, YL, and FF in spring and autumn.

| Newwork parameters | | AS | | YL | | FF | |
| --- | --- | --- | --- | --- | --- | --- | --- |
|  |  | Spring | Autumn | Spring | Autumn | Spring | Autumn |
| Number of nedos | | 182 | 202 | 239 | 265 | 266 | 313 |
| Number of edges | Bac-Bac | 75(31.78%) | 90(26.55%) | 60(14.81%) | 91(28.26%) | 479(51.89%) | 358(42.12%) |
|  | Fun-Fun | 104(44.07%) | 176(51.92%) | 257(63.46%) | 129(40.06%) | 212(22.97%) | 226(26.59%) |
|  | Bac-Fun | 57(24.15%) | 73(21.53%) | 88(21.73%) | 102(31.68%) | 232(25.14%) | 266(31.29%) |
| Average degree | | 1.297 | 1.678 | 1.695 | 1.215 | 3.47 | 2.716 |
| Average clustering coefficent | | 0.121 | 0.095 | 0.125 | 0.074 | 0.154 | 0.12 |
| Network diameter | | 6 | 6 | 6 | 5 | 8 | 9 |
| Network density | | 0.007 | 0.008 | 0.007 | 0.005 | 0.013 | 0.009 |
| Average path length | | 1.666 | 2.054 | 2.194 | 1.795 | 2.518 | 2.808 |

“Bac” means bacteria,“Fun” means fungi.

**Table S9.** Metagenomic sequences of AS phyllosphere.

| **Seasons** | **Sample ID** | **Raw base(Gb)** | **Clean** **base(Gb)** | **Q30(%)** | **Optimized base(Gb)** | **Contigs** | **Assembly Length(Gb)** | **N50(bp)** |
| --- | --- | --- | --- | --- | --- | --- | --- | --- |
| Spring | AS_2 | 7.15 | 6.95 | 93.22 | 2.86 | 240139 | 0.14 | 594 |
|  | AS_5 | 7.92 | 7.74 | 94.11 | 5.03 | 422705 | 0.29 | 706 |
|  | AS_6 | 8.53 | 8.33 | 94.19 | 6.33 | 548051 | 0.40 | 785 |
|  | AS_11 | 7.73 | 7.56 | 94.47 | 5.67 | 454143 | 0.33 | 799 |
| Autumn | AS_23 | 8.83 | 8.64 | 93.79 | 7.14 | 399195 | 0.21 | 536 |
|  | AS_32 | 7.94 | 7.56 | 94.15 | 6.37 | 538727 | 0.30 | 566 |
|  | AS_35 | 8.64 | 8.37 | 93.83 | 7.46 | 678879 | 0.42 | 624 |
|  | AS_36 | 8.46 | 8.27 | 93.94 | 7.46 | 631829 | 0.38 | 628 |
